# Opportunities and Challenges of Small Molecule Induced Targeted Protein Degradation

**DOI:** 10.3389/fcell.2021.685106

**Published:** 2021-06-22

**Authors:** Ming He, Wenxing Lv, Yu Rao

**Affiliations:** MOE Key Laboratory of Protein Sciences, School of Pharmaceutical Sciences, MOE Key Laboratory of Bioorganic Phosphorus Chemistry & Chemical Biology, Tsinghua University, Beijing, China

**Keywords:** PROTAC, protein degradation, E3 ubiquitin ligase, proteasome, small molecule inhibitor

## Abstract

Proteolysis targeting chimeras (PROTAC) represents a new type of small molecule induced protein degradation technology that has emerged in recent years. PROTAC uses bifunctional small molecules to induce ubiquitination of target proteins and utilizes intracellular proteasomes for chemical knockdown. It complements the gene editing and RNA interference for protein knockdown. Compared with small molecule inhibitors, PROTAC has shown great advantages in overcoming tumor resistance, affecting the non-enzymatic function of target proteins, degrading undruggable targets, and providing new rapid and reversible chemical knockout tools. At the same time, its challenges and problems also need to be resolved as a fast-developing newchemical biology technology.

## Introduction

**FIGURE 1:**
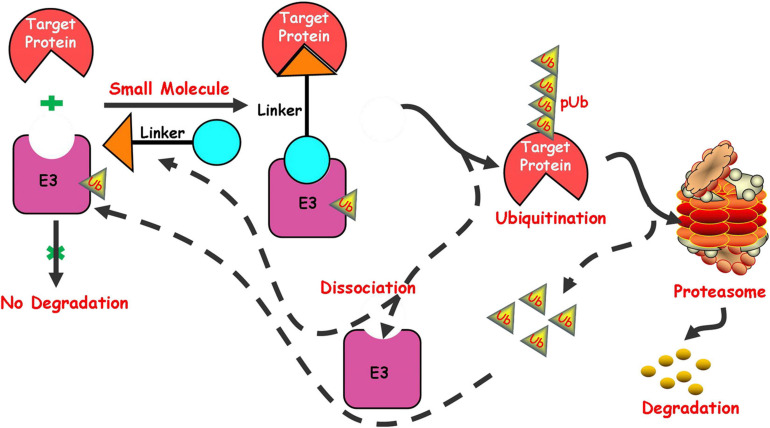
Schematic depiction of the small molecule induced protein degradation.

In recent years, targeted protein degradation has attracted much attention from the pharmaceutical industry, among which the focus is undoubtedly the proteolysis targeting chimeras (PROTAC) technology induced by small molecules. PROTAC is a new technology that uses small molecules to induce degradation of target proteins to regulate the protein level. As a new drug development strategy, its model of action is different from that of traditional small molecule inhibitors and it has a huge potentiality in overcoming drug resistance and the traditional “unable to medicine” targets (Toure and Crews, 2016; Lai and Crews, 2017; Ottis and Crews, 2017; Zou et al., 2019; Burslem and Crews, 2020). The ubiquitin-proteasome system in cell (Amm et al., 2014) plays an important role in the process of protein degradation. The system includes two main stages in the degradation of proteins. The first stage is the interaction between ubiquitin and the protein substrate to form a protein substrate-ubiquitin complex. The second stage is the degradation of the protein substrate by the proteasome, followed by the release of ubiquitin. PROTACs mainly play a related role in the first stage in the process of inducing protein degradation. PROTACs induce the target protein and E3 ubiquitin ligase to form a ternary complex by utilizing a bifunctional small molecule, which can simultaneously bind the target protein and E3 ubiquitin ligase. The complex makes the target protein recognized by E3 ubiquitin ligase and then ubiquitinated, finally recognized and degraded by the proteasome in cells (Figure 1).

**FIGURE 2:**
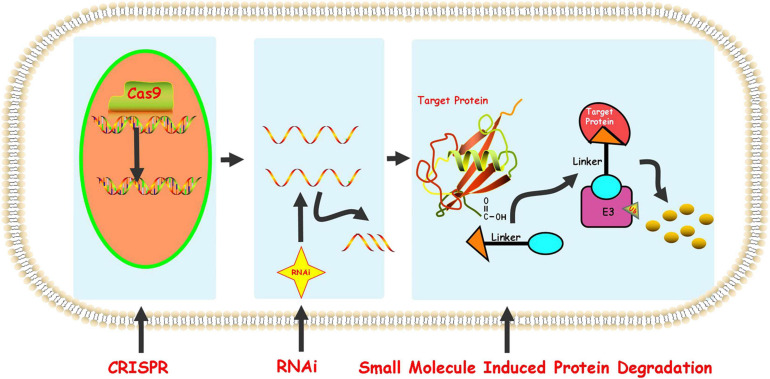
Protein knockdown strategies.

**FIGURE 3:**
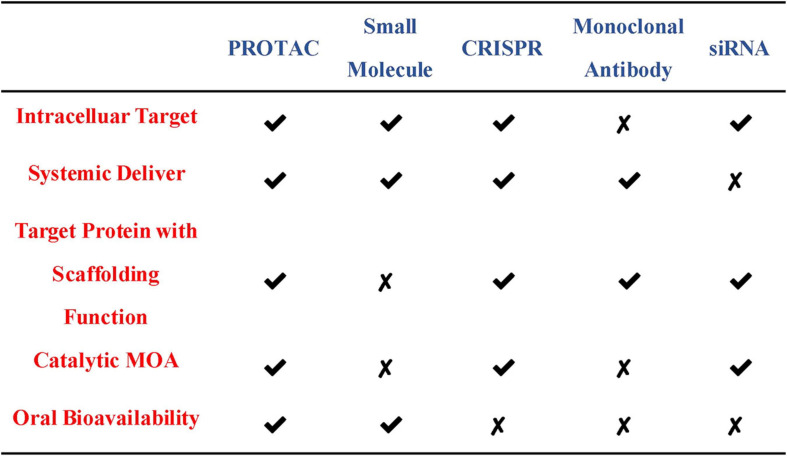
Comparisons of PROTAC with other therapeutic modalities.

At present, the regulation of the level of the target protein in cells can be intervened from three levels. Firstly, the regulation of the target protein at the gene level-the gene editing technology (CRISPR-Cas 9) that has emerged in recent years (Ebrahimi and Hashemi, 2020). Although having the advantages of high precision and strong versatility, it cannot dynamically regulate the target protein and is irreversible so that it has a potential genetic compensation effect. Secondly, the regulation of the target protein at the RNA level-RNA interference technology shows great power and charm in inducing the specific target protein degradation to regulate the target protein level. However, this technology is less efficient and not suitable for the study of relatively stable proteins (Agrawal et al., 2003; Mello and Conte, 2004). Finally, the regulation of the target protein at the protein level-the chemical knockdown of the target protein is carried out by PROTAC. This technology has the advantages of high efficiency and reversibility, and can carry out a catalytic cycle process so that PROTAC can play a role at low doses (Burslem and Crews, 2020; Figure 2). Since PROTAC can degrade the entire protein, it can affect the non-enzymatic function of the protein. Unlike traditional small molecule inhibitors that compete with active sites and occupy the pharmacological model of action, PROTAC exerts its effect through the repeated and iterative model of action to induce the target protein degradation. So in the case of target protein mutation or low expression, PROTAC will be better tolerated than traditional small molecule inhibitors. As a new and promising technology, PROTAC shows obvious advantages compared with traditional small molecule, CRISPR and other technologies (Figure 3). Compared with small molecules, PROTAC not only has the advantages of small molecules, but also makes up for its shortcomings in target protein with scaffolding function and catalytic MOA. In addition, compared with CRISPR, monoclonal antibody and siRNA technology, PROTAC has the obvious advantage of oral bioavailability.

**FIGURE 4:**
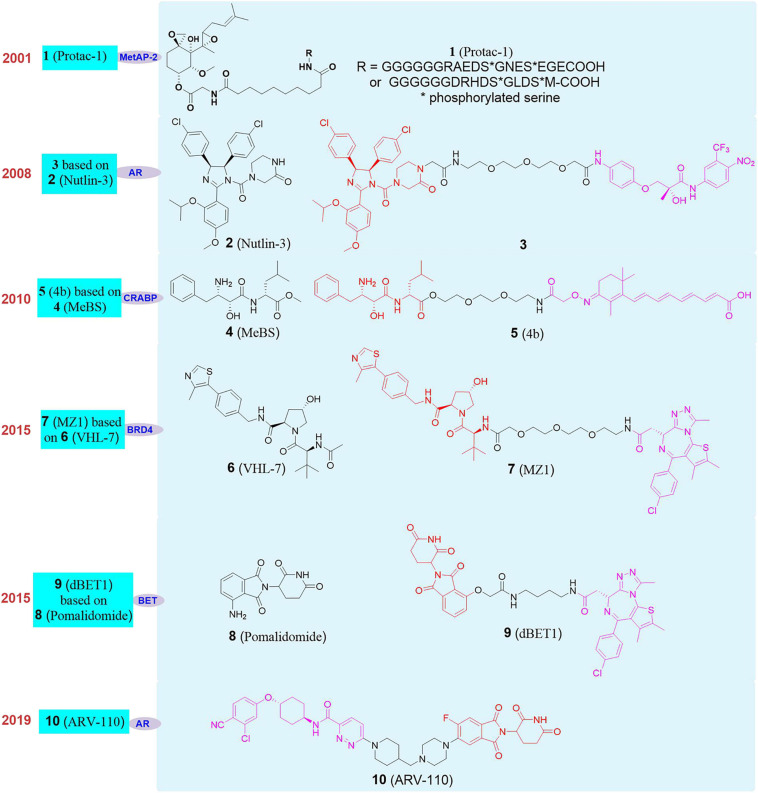
The development and structures of representative E3 ligands and PROTACs.

The concept of PROTAC was first proposed by Craig Crews in Yale University and Raymond Deshaies in California Institute of Technology in 2001. Their peptide-based **Protac-1** (**1**, Figure 4) can induce methionine aminopeptidase II (MetAP-2) degradation (Sakamoto et al., 2001). Due to the shortcomings of peptide ligand, the degradation efficiency of PROTAC was low and it developed slowly. Until 2008, Crews’s group used MDM2-p53 PPI inhibitor **Nutlin-3** (Patton et al., 2006; Tabe et al., 2007; **2**, Figure 4) as E3 ligand to design the first PROTAC based on non-polypeptide small molecule (**3**, Figure 4), and successfully achieved the effective degradation of androgen receptor (Schneekloth et al., 2008). Although the molecule had better transmembrane properties, the degradation efficiency of the androgen receptor protein was relatively average. Since then, a series of new E3 ligase ligands have been reported, which has provided an important research basis for the rapid development of PROTAC.

It was reported that Bestatin methylester (**4**, Figure 4) can bind to the cellular inhibitor of apoptosis protein 1, and induced cIAP1 to be self-ubiquitinated and degraded by the proteasome. Therefore, cIAP1 ligand was also used as an E3 ligase ligand in the design of PROTAC. Based on these researches, Hashimoto’s group reported the first degrader based on cIAP1 ligand (Itoh et al., 2010; **5**, Figure 4) for the degradation of cellular retinol and retinoic acid-binding proteins (CRABP-I and II). Subsequently, Ciulli’s group developed a series of ligands for the E3 ligase VHL (Von Hippel-Lindau), and designed the first degrader **MZ1** (Zengerle et al., 2015; **7**, Figure 4) targeting degradation of bromodomain-containing protein 4 (BRD4) based on **VHl-7** (Galdeano et al., 2014; **6**, Figure 4). At the same time, studies have found that CRBN (cereblon) is the target of immunomodulators such as thalidomide, pomalidomide, and lenalidomide. Mechanism studies have shown that combined with CRBN these drugs can recruit such as Ikaros, Aiolos, Casein kinase 1A1 (CK1α) and other substrate proteins, which trigger the ubiquitination of these proteins, then recognized and degraded by the proteasome (Fischer et al., 2014). Then, **dBET1** (Winter et al., 2015; **9**, Figure 4) as the first degrader based on Pomalidomide (**8**, Figure 4) as the E3 ligand was successfully used to degrade BET (bromodomain and extra-terminal) protein. Since then, CRBN had gradually become the most widely used E3 ubiquitin ligase in PROTAC. On the basis of the above researches, researchers have designed and synthesized a variety of PROTAC molecules and achieved the degradation of different types of target proteins in recent years. These PROTAC molecules have been used in the treatment of various diseases, including cancer, viral infections, immune disorders, and neurodegenerative diseases (Sun X. Y. et al., 2019; Zhao Q. Y. et al., 2019; Gao et al., 2020a; Sun and Rao, 2020). At present, PROTAC is developing rapidly in various fields, especially in the field of drug development. Arvinas recently announced the latest clinical data of **ARV-110** in prostate cancer patients and phase I positive efficacy data of **ARV-471** (**10**, Figure 4) in breast cancer patients. **ARV-110** is the first oral degrader and the first PROTAC drug in clinical trial that induced the degradation of androgen receptor (AR) (Neklesa et al., 2019). The data shows that **ARV-110** is safe and effective in patients with metastatic castration resistant prostate cancer (mCRPC). **ARV-471** is an another oral protein degrader developed by Arvinas based on PROTAC targeting estrogen receptor (ER). Its phase I clinical trial is mainly for adult patients with locally advanced or metastatic ER^+^/HER2^–^ breast cancer. The results show that **ARV-471** has great potential in its safety and tolerability, which is also the milestone in the transformation of PROTAC into a new treatment pattern.

The emergence of PROTAC not only opened a new chapter for new drug development, but also brought unprecedented opportunities to industry and academia. Of course, we also needed to be soberly aware that we are bound to face many new problems and challenges in the process of the rise of any new technology. It was in overcoming all kinds of difficulties that new technologies can be developed and improved, thus broadening the range of applications and influences. In recent years, our group had carried out some exploratory works in the field of small molecule induced protein degradation. The following parts will briefly summarize the main development directions and challenges of PROTAC based on the research work of our group.

## Protac Can Overcome Tumor Drug Resistance

In addition to traditional chemotherapy treatments for cancer, kinase inhibitors has developed vigorously in the past two decades. These kinase inhibitors have achieved excellent clinical effects, greatly improving the quality of life and effectively prolonging the survival period of cancer patients. However, although kinase inhibitors are very effective, patients often develop resistance to kinase inhibitors at different times, which causes disease relapse. Therefore, drug resistance caused by targeted tumor therapy is a major problem faced by cancer research.

### PROTAC Can Overcome Tumor Drug Resistance Caused by BTK Mutations

**FIGURE 5:**
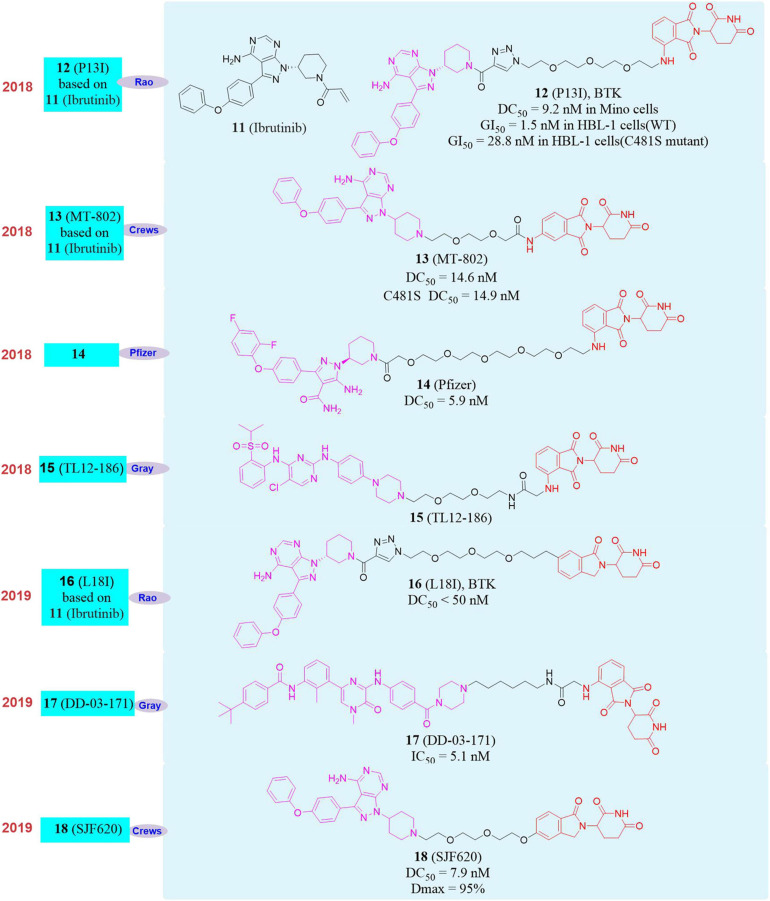
Structures of BTK inhibitor and degraders.

For example, Bruton’s tyrosine kinase (BTK) covalent inhibitor **Ibrutinib** (Pan et al., 2007; **11**, Figure 5) is used as a treatment drug for mantle cell lymphoma (Wang et al., 2013). But when it is used in clinical, **Ibrutinib** will induce C481S mutation of BTK and form drug resistance (Chiron et al., 2014; Cheng et al., 2015).

In 2018, our group reported the first new protein degrader **P13I** (Sun Y. H. et al., 2018; **12**, Figure 5), which can be used in a variety of B cell malignancies (mantle cell lymphoma, diffuse large B cell lymphoma, multiple myeloma) with drug concentration less than 10 nM to efficiently degrade BTK protein. The inhibitory activity of **P13I** on BTK-dependent wild-type human B-cell lymphoma (HBL-1) cells was equivalent to that of **Ibrutinib**. More importantly, **P13I** can induce the degradation of C481S mutant BTK protein and overcome the resistance of B-cell malignant tumor BTK kinase to **Ibrutinib** caused by the C481S mutation. And the selectivity of **P13I** was better than that of **Ibrutinib**. Even at high concentrations, it had no degradation or inhibitory activity for the targets that caused serious side effects of **Ibrutinib** (EGFR, ITK, TEC, etc.), which means that the BTK protein degrader will have better security.

Almost at the same time, Crews’s group reported another BTK degrader **MT-802** based on **Ibrutinib** (Buhimschi et al., 2018; **13**, Figure 5). **MT-802** can effectively induce the degradation of wild-type BTK protein with DC_50_ = 14.6 nM as well as the C481S mutant BTK protein with DC_50_ = 14.9 nM. And **MT-802** can reduce phosphorylation of BTK protein in tumor cells isolated from patients with C481S mutant chronic lymphocytic leukemia (CLL), while **Ibrutinib** cannot. *Pfizer* also reported the PROTAC molecule **14** (Zorba et al., 2018; Figure 5) targeting the degradation of BTK in 2018. It can efficiently degrade BTK protein after 24 h of treatment of Ramos cells, and the DC_50_ is 5.9 nM. They found that the molecule can effectively degrade the BTK protein in the rat lung and spleen *in vivo* activity evaluation, but this research did not report the degradation of the mutant BTK protein by the degrader. In the same year, Nathanael S. Gray’s group reported a multi-target degrader **TL12-186** (Huang et al., 2018; **15**, Figure 5). They found that **TL12-186** can down-regulate the expression levels of 28 kinases through quantitative proteomics research, including BTK.

In 2019, our group constructed a new framework of PROTAC molecule **L18I** (**16**, Figure 5) to target the degradation of BTK protein, which can efficiently induce multiple mutation types (C481S/T/A/G/W) BTK protein degradation in transfected HeLa cells at drug concentration less than 50 nM (Sun Y. H. et al., 2019). And more importantly, **L18I** can cause the decrease of BTK protein level in mutant diffuse large B-cell lymphoma (DLBCL) tumor, thereby effectively inhibiting the growth of the tumor *in vivo* and overcoming the tumor resistance to **Ibrutinib** caused by the C481 mutation of BTK. In the same year, Nathanael S. Gray’s group reported the PROTAC molecule **DD-03-171** (Dobrovolsky et al., 2019; **17**, Figure 5) that induced the degradation of BTK. **DD-03-171** had an effect on mantle cell lymphoma (MCL) and showed a stronger anti-proliferation inhibitory effect with IC_50_ was 5.1 nM *in vitro*. **DD-03-171** was also effective for mouse xenograft models of patient-derived tumor cells. At the same time, Crews’s group reported degrader **SJF620** (**18**, Figure 5) based on **MT-802** that can induce the degradation of BTK and had better pharmacokinetic properties. Compared with the **MT-802** molecule, **SJF620** had a longer half-life, the plasma clearance rate per unit time was lower and the absorption was better (Figueroa et al., 2020).

### PROTAC Can Overcome Tumor Drug Resistance Caused by CDK4/6 Mutations

**FIGURE 6:**
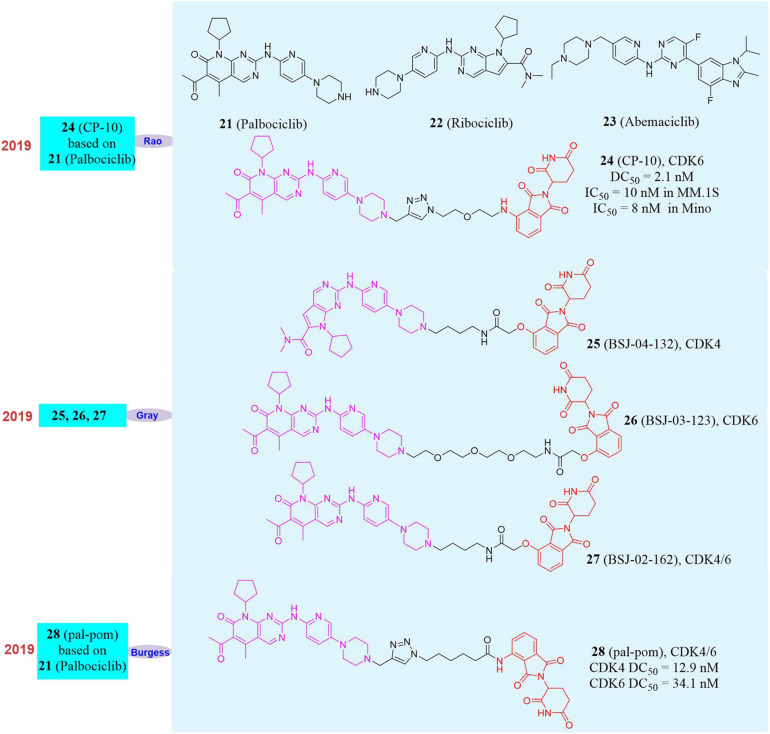
Structures of CDK4/6 inhibitors and degraders.

Cyclin dependent kinase 4/6 (CDK4/6), an important part of the cyclin family, plays a regulatory role in the transition from the G1 phase to the S phase in the cell cycle (Vermeulen et al., 2003). There are mutations or over-activation of CDK4/6 in a variety of cancer tissues (Otto and Sicinski, 2017), so CDK4/6 has always been regarded as an important target for drug development (Niesvizky et al., 2015; Tadesse et al., 2015). Numerous CDK6 inhibitors have been approved for clinical trials, among which **Palbociclib** (**21**, Figure 6) has been approved for the treatment of estrogen receptor-positive breast cancer patients (Leonard et al., 2012; Barton et al., 2013). However, because CDK6 point mutations may weaken drug binding affinity and form drug resistance (Yang et al., 2017; Li et al., 2018), the development of traditional small molecule inhibitors of CDK6 is extremely difficult. Therefore, new chemical biological methods to develop a small molecule drug targeting CDK6 has become a practical strategy for the treatment of malignant tumors.

Until now, three FDA-approved CDK4/6 inhibitors **Palbociclib**, **Ribociclib** (**22**, Figure 6) and **Abemaciclib** (**23**, Figure 6) have strong affinity to CDK6, but the binding models are different (Su et al., 2019). Based on these inhibitors, our group developed a series of small molecules degraders. Subsequently, the activity of all small molecules degrading CDK6 *in vitro* was evaluated. The compound **CP-10** (**24**, Figure 6) was found to have the best CDK6 degradation activity. In human glioblastoma U251 cells, the degradation of CDK6 induced by **CP-10** (DC_50_ = 2.1 nM) was nearly 72% at 10 nM and 89% at 100 nM. The degradation of CDK4 induced by **CP-10** was much weaker than that of CDK6 (DC_50_ was approximately 50–80 times more than that of CDK6). It was found that the degradation effect was dependent on the length of the linker during the evaluation of CDK6 degradation activity. PROTAC with a shorter linker had a higher degradation ability, which mean that these shorter molecules had a better ability to recruit CRBN to CDK6. At the same time, **CP-10** can also induce the degradation of CDK6*^*D*163G^* and CDK6*^*S*178*P*^* mutations. Compared with the wild-type, the degradation of CDK6*^*D*163G^* was slightly weakened, but still remarkable, indicating that although the affinity of **Palbociclib** in the **CP-10** molecule was destroyed due to the mutation of the binding site, it was sufficient to induce CDK6 degradation. These data indicated that small molecules induce CDK4/6 degradation had good application and potential in overcoming **Palbociclib** resistance. In addition, **CP-10** (IC_50_ = 10 nM) showed a better inhibitory effect than **Palbociclib** (IC_50_ = 200 nM) in multiple myeloma cells (MM.1S cells), and the anti-proliferative activity of **CP-10** (IC_50_ = 8 nM) was also better than that of **Palbociclib** (IC_50_ = 45 nM) in mantle cell lymphoma cells (Mino cells), and it had comparable activity in leukemia cells. Interestingly, we found that the CDK6 protein was only degraded when PROTAC recruits CRBN, but not when the other three E3 ligase (VHL/MDM2/cIAP) ligands were used.

At the same time, Gray’s group had successively reported different degraders for CDK4, CDK6, and CDK4/6. They used **Ribociclib**, **Palbociclib,** and pomalidomide as ligands to achieve the selective degradation of CDK4/6 by changing the type of linker (Brand et al., 2019; Jiang et al., 2019). **BSJ-04-132** (**25**, Figure 6) can selectively induce CDK4 degradation, **BSJ-03-123** (**26**, Figure 6) can selectively induce CDK6 degradation, and **BSJ-02-162** (**27**, Figure 6) can simultaneously induce CDK4/6 degradation. Compared with CDK4/6 inhibitors, these degraders can show stronger protein degradation ability at 100 nM and better anti-tumor cell proliferation activity than inhibitors. In addition, the degrader **BSJ-02-162** in the human mantle cell lymphoma cell line (Granta-519) may cause significant degradation of CDK4/6 and at the same time induce tumor cell G1 cell cycle arrest. Burgess’s group also reported the CDK4/6 degrader **pal-pom** (**28**, Figure 6) which also used **Palbociclib** and pomalidomide as ligands (Zhao and Burgess, 2019), and its difference to **CP-10** was only on the linker. They found that the compound **pal-pom** showed better degradation activity to CDK4 than that to CDK6, and its DC_50_ to CDK4 and CDK6 were 12.9 and 34.1 nM, respectively. Subsequently, the research group tested its anti-tumor cell growth activity and found that the degrader had a poor tumor suppressing activity when the IC_50_ was at 10–50 μM in MDA-MB-231 cells.

### PROTAC Can Overcome Tumor Drug Resistance Caused by BCR-ABL Mutations

**FIGURE 7:**
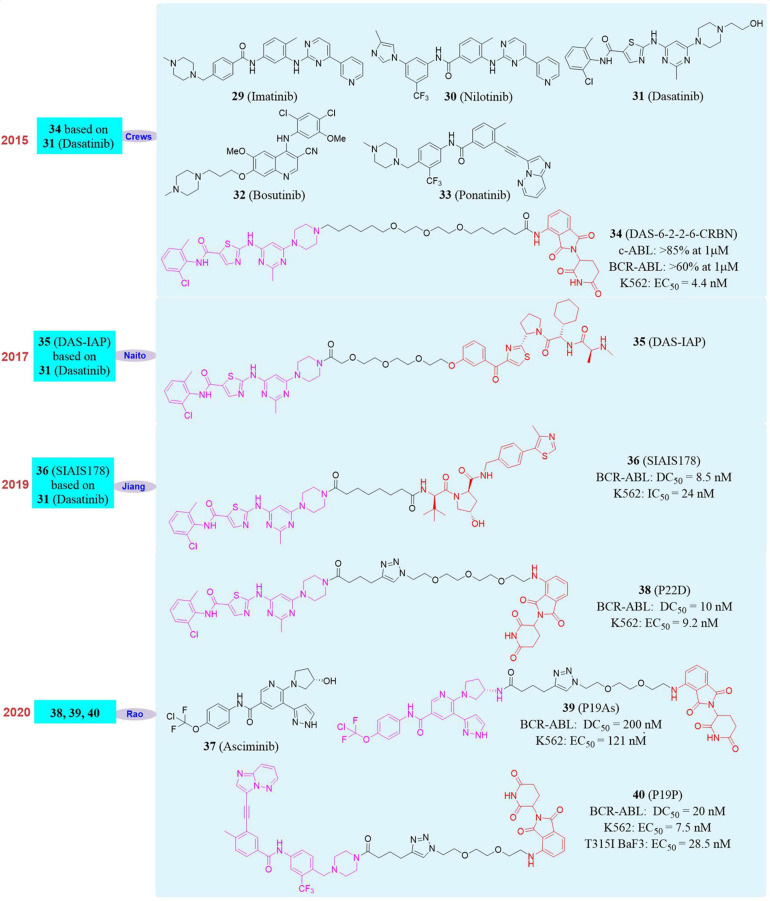
Structures of BCR-ABL inhibitors and degraders.

The BCR gene is located on chromosome 22 and the normal BCR gene product is cytosolic phosphoprotein. ABL is a proto-oncogene located on chromosome 9 and the gene product is a non-receptor tyrosine protein kinase, which plays an important role in cell differentiation and cell cycle regulation in normal cells (Talpaz et al., 2006). The chromosomal translocation of t(9; 22) (q34; q11) will lead to the formation of BCR-ABL fusion gene and the gene product is BCR-ABL fusion protein. Its expression leads to the activation of ABL tyrosine kinase, changes of the cell’s tyrosine protein level and capacity of actin binding, which disrupts the normal signal transduction pathway and inhibits the occurrence of apoptosis (Pophali and Patnaik, 2016). Positive (Ph+) acute lymphoblastic leukemia (ALL) and more than 95% of chronic myelogenous leukemia (CML) patients have the BCR-ABL fusion gene. The current new drug for BCR-ABL tyrosine kinase treatment of CML is still ATP competitive inhibitors (Yang and Fu, 2015). **Imatinib** (**29**, Figure 7), as the first tyrosine kinase inhibitor (TKI) and the first-generation ABL inhibitor, has achieved significant clinical effects and is used as a first-line treatment for CML patients. Although a model of targeted cancer therapy, **Imatinib** is ineffective for about 40% patients due to intolerance and drug resistance because of BCR-ABL mutation, especially the T315I mutation, which makes patients form more serious resistance. The advent of the second-generation ABL inhibitors **Nilotinib** (**30**, Figure 7), **Dasatinib** (**31**, Figure 7) and **Bosutinib** (**32**, Figure 7) and the third-generation ABL inhibitors **Ponatinib** (**33**, Figure 7) have provided multiple treatment options to patients with mutations. However, the new TKI can not inhibit all resistant mutants and will induce such side effects as vascular disease, which severely limits their clinical use. **Ponatinib**, as the only drug that targets the BCR-ABL T315I mutation, was temporarily delisted in 2013 due to its serious vascular adverse events, and was later approved for use in the revised indication (Lu et al., 2015). So far, there are no new drugs approved for targeted therapy of T315I mutants, so the design and development of BCR-ABL degrader based on PROTAC is of great significance.

In 2016, Crews’s group developed the first BCR-ABL degrader (Lai et al., 2016). They constructed the PROTAC molecule **DAS-6-2-2-6-CRBN** (**34**, Figure 7) based on CRBN and **Dasatinib** to induce c-ABL degradation. After evaluation, **DAS-6-2-2-6-CRBN** derived from **Dasatinib** caused the degradation of two types of ABL proteins. The degradation effects were c-ABL (>85% at 1 μM) and BCR-ABL (>60% at 1 μM). At the same time, they also found that **DAS-6-2-2-6-CRBN** had a significant inhibitory effect on the growth of K562 cells, with an EC_50_ of 4.4 nM. Subsequently, in 2017, Naito’s group reported the second **Dasatinib**-derived BCR-ABL PROTAC molecule **DAS-IAP** (**35**, Figure 7), which had good activity in inhibiting the growth of CML cells and sustaining anti-proliferation effects (Shimokawa et al., 2017). In 2019, Biao Jiang’s group reported the first PROTAC molecule **SIAIS178** (**36**, Figure 7) based on VHL and **Dasatinib**. The **SIAIS178** had good selectivity, and its DC_50_ to BCR-ABL was 8.5 nM. It showed good anti-proliferative activity on K562 cells with IC_50_ of 24 nM, and induced *in vivo* regression of K562 xenograft tumors (Zhao Q. J. et al., 2019). Although the above studies have obtained degraders with obvious degradation effects and excellent cytostatic activity, they had no degradation effect on mutant BCR-ABL. This has become the biggest disadvantage, thus restricting their further use. Therefore, the development of degrader that can induce the degradation of wild-type and mutants BCR-ABL is particularly important.

In 2020, our group used four BCR-ABL inhibitors **Imatinib**, **Dasatinib**, **Asciminib** (**37**, Figure 7) and **Ponatinib** as target molecules (Yang Y. Q. et al., 2020). Due to the fact that they had different binding sites and binding models with BCR-ABL, they were used separately to design and synthesize a series of small degraders. Subsequently, the activity of all small degraders to induce BCR-ABL degradation *in vitro* was evaluated. It was found that the degraders designed with **Imatinib** did not induce BCR-ABL degradation, and the degraders designed based on the other three BCR-ABL inhibitors can induce BCR-ABL degradation. Especially the compound **P22D** (**38**, Figure 7, DC_50_ = 10 nM) designed based on **Dasatinib**, the compound **P19As** (**39**, Figure 7, DC_50_ = 200 nM) designed based on **Asciminib**, the compound **P19P** (**40**, Figure 7, DC_50_ = 20 nM) designed based on **Ponatinib** performed well, and also showed good cytostatic activity. In K562 cells, its anti-proliferative activity was **P22D** (EC_50_ = 9.2 nM) and **P19P** (EC_50_ = 7.5 nM), which has comparable cell proliferation activity to that of **DAS-6-2-2-6-CRBN** (EC_50_ = 8.8 nM) developed by Crews’s group. Subsequently, our group also tested the degradation effect of these BCR-ABL degraders on mutant BCR-ABL and the anti-proliferation effect on mutant cell lines. It was found that compound **P19P** has the best degradation effect on T315I mutant BCR-ABL. However, its degradation activity was significantly weaker than that of wild-type BCR-ABL. And **P19P** had better anti-proliferative activity against T315I mutant BaF3 cell line, with an EC_50_ of 28.5 nM. In addition to T315I, **P19P** can also induce the degradation of BCR-ABL with V468F and other mutants (such as E255K and H396R) in transfected HeLa cells. At the same time, it was also proved that these degraders have less side effects on the cardiovascular system and have excellent kinase selectivity.

### PROTAC Can Overcome Tumor Drug Resistance Caused by BRAF Mutations

The BRAF gene is responsible for encoding the RAF kinase protein that transmits cell signals. This protein is part of the RAS-RAF-MEK-ERK signaling pathway (the MAPK/ERK pathway) (Samatar and Poulikakos, 2014). BRAF is one of the most important proto-oncogenes in humans and about 8% of human tumors have BRAF mutations, of which the vast majority are BRAF-V600E mutation and mainly occur in melanoma, colon cancer and thyroid cancer (Holderfield et al., 2014). In normal humans, RAF kinase protein activates the phosphorylation of MEK/ERK, thereby playing a corresponding role in cell proliferation. In this signal pathway, normal human body will have corresponding feedback regulation, so that the phosphorylation of RAF kinase protein can be maintained at a normal level (Karoulia et al., 2017). However, when the RAF protein kinase is mutated, especially the V600E mutation, the feedback effect can only act on the wild-type RAF protein kinase, and there is no feedback effect on the V600E type RAF protein kinase, which leads to the continuous activation of the downstream MEK-ERK signaling pathway. It plays a vital role in tumor growth, proliferation, invasion and metastasis, so BRAF-V600E mutation is one of the effective targets of anti-melanoma and other tumors (Agianian and Gavathiotis, 2018).

**FIGURE 8:**
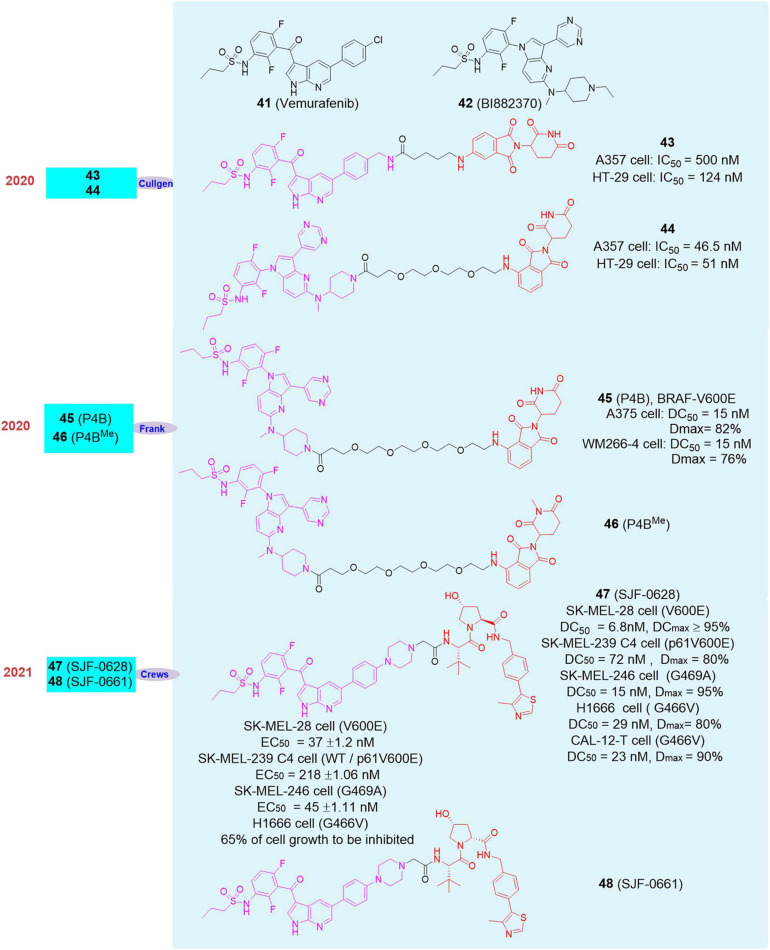
Structures of BRAF inhibitors and degraders.

In 2011, the first BRAF-V600E targeted inhibitor, **Vemurafenib** (**41**, Figure 8), was approved by the FDA for the treatment of patients with BRAF-V600E mutations in advanced melanoma and achieved breakthrough therapeutic effects (Bollag et al., 2012). **Vemurafenib** is also a typical target drug based on genetic diagnosis. However, it is reported that after 6–12 months of taking **Vemurafenib**, patients will have varying degrees of drug resistance, which limits the therapeutic effect (Lito et al., 2013). Therefore, the development of new small-molecule drugs targeting BRAF-V600E mutations is particularly important.

In 2020, Cullgen selected the BRAF-V600E mutation-targeting inhibitor **Vemurafenib** and the pan-inhibitor **BI882370** (**42**, Figure 8) as the BRAF-V600E ligands (Han et al., 2020). By analyzing the binding model of small molecule inhibitors and proteins, they designed a series of degraders, finding that compound **43** (Figure 8) based on **Vemurafenib** and compound **44** (Figure 8) based on **BI882370** had better activity to degrade BRAF-V600E protein. They also found that compound **43** can induce the degradation BRAF-V600E protein at 12 nM, and the degradation effect was significantly enhanced with the increase of the drug dose; while compound **44** had a degradation effect at 37 nM, and it also showed dose-dependent effect. In order to prove that the degraders had no degrading activity on wild-type BRAF protein, they used compound **43** and compound **44** to perform protein degradation experiments on A549 cells (BRAF-WT), and they found that these two degraders had no degradation activity on the BRAF-WT protein at different concentrations, which proved that the degraders had an excellent selective degradation effect on the BRAF-V600E protein. Finally, in order to prove the anti-tumor effect, they used compounds **43** and **44** to do anti-proliferation experiments on cell lines A375 and HT-29, respectively. The experimental results showed that compound **43** based on **Vemurafenib** had a worse inhibitory effect on A375 cells than **Vemurafenib**, but the effect was adverse on the cell line HT-29, with an IC_50_ of 124 nM. While compound **44** based on **BI882370** had the same inhibitory effect on the cell line A375 and HT-29, with IC_50_ of 46.5 and 51 nM, respectively.

In the same year, Frank Sicheri’s group reported the BRAF-V600E degrader. Based on the screening of E3 ligase binders, linkers and BRAF binders, they obtained the compound **P4B** (**45**, Figure 8) with the best degradation effect and the negative control **P4B^Me^** (**46**, Figure 8; Posternak et al., 2020). In subsequent experiments, they found that in A375 cells, the D_max_ of **P4B** to BRAF-V600E was 82%, and the DC_50_ was 15 nM. At a higher concentration (DC_50_ = 1000 nM), **P4B** did not reduce the level of ARAF, but slightly decreased CRAF level (D_max_ = 20–45%) and had no effect on the level of RAF family members KSR1 or SRMS. These results were also seen in SK-MEL-28 cells. Next, they tested the anti-proliferative effects of **P4B**, **P4B^Me^** and inhibitor **BI882370** on cells. Compared with **P4B^Me^** and **BI882370**, **P4B** showed excellent anti-proliferative activity. In homozygous A375 cells (V600E), its inhibitory activity was significantly better than that of heterozygous COLO-205 cells (BRAF WT/V600E)/RKO cells (BRAF WT/V600E) and HT-29 cells (BRAF WT/V600E/V600E). Considering that **P4B** had comparable enzymatic activity to BRAF-WT and BRAF-V600E *in vitro* (IC_50_ = 58 nM *vs.* 12 nM), it showed that **P4B** had good degradation selectivity to BRAF-V600E in cells. At the same time, they also found that **P4B** had a certain degradation effect on other BRAF mutants. In the melanoma cell line WM266-4 (V600D), the degrader showed strong degradation activity with DC_50_ and D_max_ of 15 nM and 76%, respectively. At high concentrations, **P4B** had a weaker proliferation inhibitory effect on the NCI-H1666 cell line with heterozygous BRAF-G466V mutation, and had no effect on the H508 (G596R) and NCI-H1755 cell lines (G469A).

In 2021, Crews’s group reported the degrader **SJF-0628** (**47**, Figure 8) and the negative control **SJF-0661** (**48**, Figure 8) based on **Vemurafenib** and VHL that target multiple BRAF mutations (Alabi et al., 2021). In their research, they found that the degrader can induce the degradation of BRAF-V600E protein in a variety of cell lines, without inducing the degradation of BRAF-WT protein. In SK-MEL-28 cells, the DC_50_ to BRAF-V600E was 6.8 nM, and the DC_max_ was more than 95%. The same phenomenon can be observed in A375 cells and SK-MEL-239 cells. The degrader can completely induce the degradation of BRAF-V600E within 4 h (the drug concentration was 100 nM), its degradation of BRAF-V600E and inhibition of *p*-ERK lasted up to 72 h. In the wash out experiment, the BRAF level recovered 30% after 24 h. At the same time, they also found that **SJF-0628** can inhibit the phosphorylation of MEK and ERK in SK-MEL-28 cells at 10 nM. The degrader **SJF-0628** not only can induce the degradation of BRAF-V600E, it can also induce the degradation of a variety of BRAF mutants in a variety of cell lines. In SK-MEL-239 C4 cells, they found that the degrader can induce the degradation of BRAF-p61^V600E^ with DC_50_ and D_max_ of 72 nM and 80%, and had no effect on BRAF-WT and CRAF. In SK-MEL-246 cells, it can induce the degradation of BRAF-G469A, and its DC_50_ and D_max_ were 15 nM and 95%, respectively. In H1666 and CAL-12-T, it can induce the degradation of BRAF-G466V, and its DC_50_ and D_max_ were DC_50_ of 29 nM, D_max_ of 80% and DC_50_ of 23 nM, D_max_ of 90%, respectively. Subsequently, they tested the inhibitory effect of the degrader **SJF-0628** on the tumor cells. They found that in SK-MEL-28 cells (BRAF-V600E), the EC_50_ of **Verofenil** and the negative control **SJF-0661** were 215 ± 1.09 nM and 243 ± 1.09 nM, respectively, while the EC_50_ of the degrader **SJF-0628** was 37 ± 1.2 nM. Although these compounds had comparable binding capacity to BRAF-V600E *in vitro* (**Vemurafenib** = 27 nM, **SJF-0628** = 39 nM, **SJF-0661** = 64 nM), **SJF-0628** had significantly stronger anti-tumor activity. In SK-MEL-239 C4 cells (BRAF-WT/BRAF-p61^V600E^), **Vemurafenib** and **SJF-0661** had weaker growth inhibitory effect compared with **SJF-0628**, which can reduce about 80% of a cell growth, and its EC_50_ was 218 nM ± 1.06. In SK-MEL-246 cells (BRAF-G469A), **SJF-0628** can also effectively inhibit cell growth, with an EC_50_ of 45 ± 1.11 nM, and the EC_50_ of the negative control **SJF-0661** was 278 ± 1.07 nM. In H1666 cells (BRAF-G466V), **SJF-0628** can induce 65% of cell growth to be inhibited, while the inhibitory effect of **Verafenib** was less than 50%. These results showed that targeted degradation can be used to overcome acquired resistance to BRAF inhibitor-based therapies.

In summary, the small molecules induced degradation of BTK, CDK4/6, BCR-ABL and BRAF in cancer cells had a better anti-tumor cell proliferation effect than simply inhibiting, and they also demonstrated promising power in overcoming tumor drug resistance. These research results showed the degradation of protein to overcome drug resistance has good potential and they also increase the potential application of PROTACs in clinical practice.

## Protac Can Induce the Entire Protein Degradation to Affect Non-Enzymatic Functions

Traditional small-molecule drugs usually act by inhibiting the enzymatic function of the target and have no effect on the non-enzymatic function, while PROTAC can induce the entire protein degradation, so PROTAC can affect the protein’s enzymatic function and regulate the non-enzymatic function.

Focal adhesion kinase (FAK), also known as protein tyrosine kinase 2 (PTK2), has kinase-dependent enzyme activity functions and kinase-independent backbone functions. These two functions play a vital role in tumors genesis, early embryonic development, and reproduction (Serrels et al., 2007; He et al., 2012; Hartman et al., 2013; Brami-Cherrier et al., 2014; Cromm et al., 2018). Although some FAK small molecule inhibitors have been clinically tested in a variety of malignant tumors, the non-enzymatic functions of FAK still cannot be blocked by reported FAK kinase inhibitors. Because traditional kinase inhibitors can only act on protein kinase domains, drug resistance is likely to occur. Therefore, developing a strategy that can inhibit FAK kinase activity and block its non-kinase activity is an urgent and meaningful need for FAK-related diseases.

**FIGURE 9:**
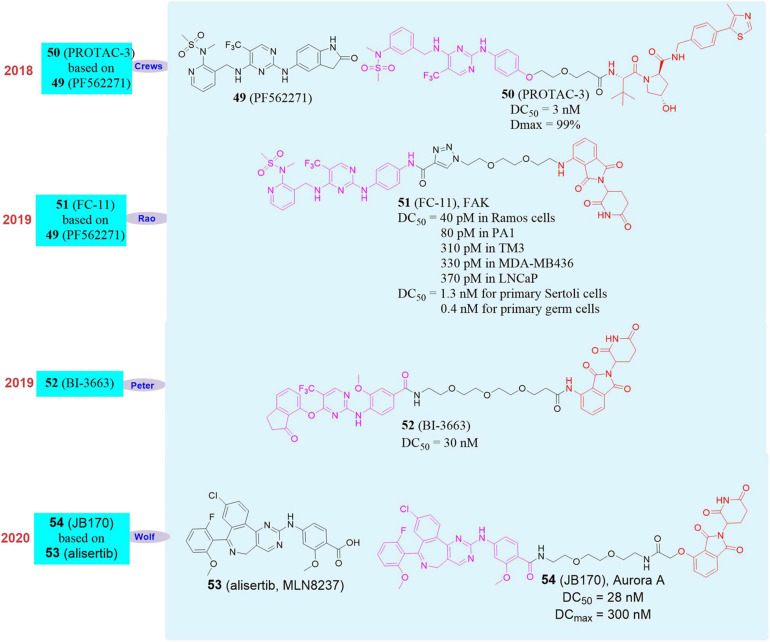
Structures of FAK and Aurora A inhibitors and degraders.

In 2018, Crews’s group reported the first PROTAC molecule **PROTAC-3** (**50**, Figure 9) that induced the degradation of FAK (Cromm et al., 2018). At the cellular level, this molecule can induce FAK degradation at nanomolar levels on a variety of cell lines. The PROTAC molecule performed better than inhibitor in the downstream signaling pathways of FAK (*p*-FAK, *p*-Paxillian, *p*-Akt) and in the invasion and migration of human triple-negative breast cancer cell (MDA-MB-231).

In 2019, our group used PROTAC to successfully obtain an efficient, rapid and reversible PROTAC molecule **FC-11** (Gao et al., 2019; **51**, Figure 9) that targets FAK and can induce FAK protein degradation in the tested cells. Its FAK protein degradation activity in the tested cells can reach to less than 1 μM and the DC_50_ can reach to 80 pM when it has been used on PA1 cells for 8 h. In the same year, Peter Ettmayer’s group developed the PROTAC molecule **BI-3663** (**52**, Figure 9) that induced FAK degradation (Popow et al., 2019). **BI-3663** efficiently induced FAK degradation with DC_50_ = 30 nM in 11 human hepatocellular carcinoma cell lines. Although the compound can effectively induce FAK degradation, it basically did not affect the cell proliferation activity of any tested cell line. In 2020, our group conducted the research on the non-enzymatic function of FAK based on **FC-11**. First of all, under the same action time and concentration, the degradation activity of **FC-11** on activated FAK (*p*FAKtyr397) was much better than that of small molecule inhibitor **PF562271** (**49**, Figure 9). In the mouse model, **FC-11** can efficiently induce FAK degradation in the mouse reproductive system, and the FAK in the mouse reproductive system can return to normal levels in 2 weeks after stopping the administration. Compared with the FAK inhibitor **PF562271**, the sperm motility and motility of mice in the **FC-11** administration group were significantly reduced, which in turn led to the decrease of fertility and maldevelopment of embryos in mice (Gao et al., 2020b). In short, **FC-11** can induce FAK degradation efficiently and reversibly, thereby affecting the non-enzymatic function of FAK.

The cycle of eukaryotic cells is not only controlled by CDKs, but also affected by other kinases (Nigg, 2001). Aurora A kinase (Aurora kinase A), which is an important subtype in the Aurora kinase family, plays an important role in phosphorylating a variety of proteins during mitosis and its catalytic activity is essential for the entire cell cycle (Marumoto et al., 2005). As scientists studied Aurora kinase, it was discovered that Aurora A kinase activity can be divided into enzymatic activity and non-enzymatic activity (Otto et al., 2009). The inhibition or consumption of Aurora A enzymatic activity may be lethal because it will cause oncogene activation or the loss of tumor suppressor genes. Its non-enzymatic function enables Aurora A to bind to the proto-oncoproteins of the MYC family and protects N-MYC and C-MYC from proteasome degradation, which has nothing to do with the enzymatic activity (Toya et al., 2011; Brockmann et al., 2013; Dauch et al., 2016). Studies have found that Aurora A kinase inhibitors may not eliminate all carcinogenic activities of Aurora A, but whether this effect is related to the non-enzymatic effect of Aurora A kinase is not yet known (Zheng et al., 2016).

In 2020, Elmar Wolf’s group selected the Aurora A kinase inhibitor **alisertib** (**53**, MLN8237, Figure 9) and designed a series of degraders that target Aurora A by linking **alisertib** with CRBN and VHL (Adhikari et al., 2020). They found that the compound **JB170** (**54,** Figure 9) had strong binding ability and degradation activity to Aurora A kinase. The experimental results proved that the DC_max_ (maximal degradation concentration) and DC_50_ of **JB170** to Aurora A kinase were 300 and 28 nM, respectively. Subsequently, they analyzed the affinity of **alisertib** (Aurora A EC_50_ = 18 nM, Aurora B EC_50_ = 51 nM) and **JB170** (Aurora A EC_50_ = 193 nM, Aurora B EC_50_ = 1.4 μM), which showed the affinity of **JB170** to Aurora A was better than that to Aurora B. After MV4-11 cells were treated with **JB170** or **alisertib**, **JB170** reduced Aurora A levels by 73%, which was 57% lower than that of **alisertib**. Among the 4259 detectable proteins, no other proteins were down-regulated, including Aurora B. At the same time, they also confirmed the selective degradation of Aurora A by **JB170** through siRNA.

It is currently known that the enzyme activity of Aurora A is mainly expressed in the G2/M phase of the cell cycle, and its function in the S phase may not be related to its enzymatic activity. Therefore, they analyzed the effect of **JB170** and **alisertib** on the cell cycle of MV4-11 cells. The experimental results showed that almost all cells were blocked in G2/M after 12 h treatment with **alisertib**. On the contrary, the treatment of **JB170** can hardly caused cell accumulation in G2/M phase, but can delay the progress of S phase. RNA sequencing showed that **alisertib** induced the expression of genes that indicate cell G2/M phase arrest, while **JB170** did not activate the same genes. In order to study the effect of **JB170**-mediated degradation of Aurora A on the survival of cancer cells, they used **JB170** (1 μM) to treat MV4-11 cells to measure cell viability. After 72 h, the number of viable cells was 32% of the control level, and similar results were observed in the colony formation test using IMR5 cells. Therefore, they believed that the arresting effect of **JB170** in the cell’s S-phase was mainly caused by the effect of Aurora A non-enzymatic activity, which laid a solid foundation for further understanding of the function of Aurora A and the development of drugs for Aurora A kinase.

Therefore, these results indicate that PROTAC can affect the non-enzymatic function of the protein, thus expanding the drug-forming possibility of existing drug targets.

## Protac Is Expected to Induce the Degradation of Undruggable Targets

As we all know, only 20–25% of the currently known protein targets are used for drug discovery and related disease treatment, including kinases, GPCRs, nuclear hormone receptors, iron channels, *etc*. The physiological significance and the relationship with disease of the remaining proteins are still to be explored, so the development of new drug targets is getting more and more attention.

### PROTAC Can Induce the Degradation of STAT3

One of the most challenging targets is the signal transducer and activator of transcription 3 (STAT3). STAT3 plays an extremely important role in cell growth, reproduction, apoptosis, metabolism, drug resistance and other processes. It is responsible for transmitting signals from the cell surface receptors to the nucleus. The continuous activation of STAT3 is often associated with the poor prognosis of human cancer, because the activated STAT3 signal can not only promote the growth, survival and metastasis of tumor cells, but also inhibit the anti-tumor immune response, STAT3 is an attractive target for the treatment of human cancer and other diseases (Takeda et al., 1997; Boccaccio et al., 1998; McLemore et al., 2001). Although scientists have been working tirelessly on this target for 20 years, targeting STAT3 is still a huge challenge. Due to the conservative structure and low specificity of STAT3, it is extremely difficult to develop efficient and specific inhibitors. Although some STAT3 inhibitors with anticancer activity have been reported so far, most of the inhibitors have poor activity and lack specificity. At present, most studies on STAT3 inhibitors are based on the SH2 domain on the STAT3 protein. The SH2 domain plays a key role in the dimerization of STAT3. Therefore, drugs targeting the SH2 domain can inhibit the dimerization of STAT3, thus inhibiting its transcriptional activity.

**FIGURE 10:**
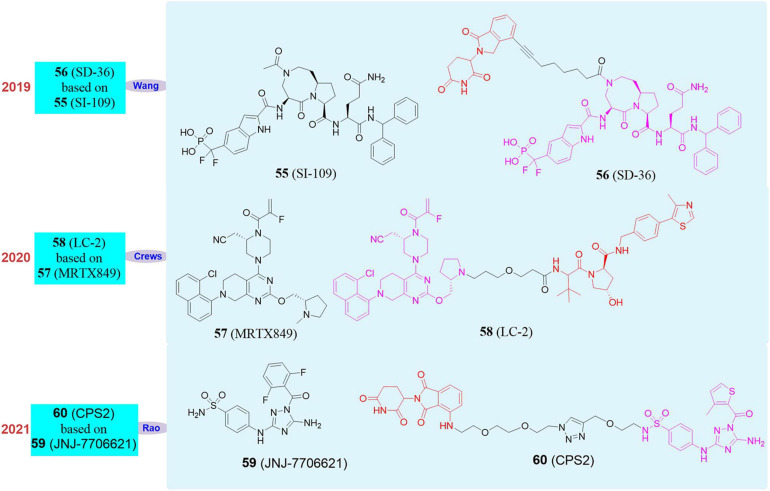
Structures of STAT3, KRAS^G12C^ and CDK2 inhibitors and degraders.

In 2019, Wang Shaomeng’s group developed an SH2 inhibitor **SI-109** (**55**, Figure 10), and successfully screened out the first degrader **SD-36** (**56**, Figure 10) that can target STAT3 by using PROTAC (Bai et al., 2019). This molecule had a high specificity for STAT3 binding and no obvious effect on other members of the STAT family. At the same time, **SD-36** still showed a good effect on the mutant SH2, so even if STAT3 had a high mutation rate, it can still be effectively degraded by **SD-36**. **SD-36** also had shown good efficacy data on leukemia model mice and can eliminate tumors lastingly and almost completely.

### PROTAC Can Induce the Degradation of KRAS^G12*C*^

Another challenging target is KRAS. KRAS is one of the most frequently mutated oncogenes, which can be activated through a variety of ways and cause tumor development, such as binding to guanosine triphosphate and activated by cell surface receptors. In addition to protein-based activation, KRAS can also be activated due to mutations in key codons. Clinically high-frequency mutations (such as G12A, G12C, G12D, G12S, G12V, G13C, G13D) and some low-frequency mutations can both activate KRAS. The mutated codon interacts with guanosine triphosphate hydrolase, which activates KRAS and ultimately leads to tumorigenesis. KRAS has mutations in a variety of cancers, among which the mutation rate of pancreatic cancer is as high as 90%, that of colon cancer and lung cancer (mostly non-small cell lung cancer) account for 30–50% and 19% respectively, and cholangiocarcinoma accounts for about 26%. Mutations also occur in cancers such as small bowel cancer, skin cancer, bladder cancer and breast cancer (Pylayeva-Gupta et al., 2011; Rojas et al., 2012; Lu et al., 2016; Cagir and Azmi, 2019; Kessler et al., 2019). Since the KRAS protein has no suitable binding pockets for small molecule inhibitors, the development of small molecule drugs targeting KRAS has not made major breakthroughs for a long time. Although the research on covalent inhibitors of KRAS*^G12*C*^* mutants has been spotlighted in recent years and there have been many KRAS*^G12*C*^* inhibitors in the clinical research stage, the clinical results also show that some patients have already developed drug resistance. However, the PROTAC has unique advantages in such difficult-to-target drug targets.

In 2020, Crews’s group reported the PROTAC molecule **LC-2** (**58**, Figure 10; Bond et al., 2020) based on the KRAS*^G12*C*^* inhibitor **MRTX849** (**57**, Figure 10; Hallin et al., 2020). **LC-2** can rapidly degrade KRAS*^G12*C*^* in different homozygous and heterozygous tumor cells. In NHC-H2030 cells, the DC_50_ of **LC-2** to KRAS*^G12*C*^* was 0.59 μM. Since **LC-2** was still covalently bound to the target protein, it would affect the catalytic cycle of the PROTAC molecule and may therefore limit its effectiveness. Although there are limitations, the appearance of **LC-2** degrader creates new opportunities for targeting KRAS mutants in cancer treatment, which is of great significance. Therefore, it is imperative to develop reversible PROTAC against KRAS mutants.

### PROTAC Can Induce the Selective Degradation of CDK2

Cyclin-dependent kinases (CDK) are a group of serine/threonine protein kinases with a shorter N-terminus (β-sheet) and a longer C-terminus (α-helix). CDK, who interacts with the corresponding cyclin and cyclin-dependent kinase activated kinase (CAK), plays a significant role in all stages of the cell cycle and participates in the physiological processes of cell growth, differentiation, and proliferation. CDK2 is a member of the CDK family and is widely expressed in mammalian cells. Because its function can be compensated by other CDK family members, CDK2 is not necessary for most normal cells and tissues. However, CDK2 plays an important role in tumorigenesis, cell differentiation, meiosis, and hearing damage repair (Ying et al., 2018). Recent studies have found that CDK2 knockdown can induce differentiation of AML cells (Takada et al., 2017). However, there is no good tool molecule that can successfully knockdown CDK2 selectively because traditional small molecule inhibitors are difficult to achieve selective CDK2 inhibition and they can not eliminate the non-enzymatic function of CDK2 (Tadesse et al., 2019). Gene editing technology has many shortcomings in effective application and clinical use. Although CDK2 is considered a good drug target, it is difficult for existing tools to achieve efficient and selective regulation.

In 2021, our group reported that we used pan-CDK inhibitor **JNJ-7706621** (**59**, Figure 10) and pomalidomide as ligands to design a new type of CDK2 selective degrader **2** (**60**, Figure 10), which achieved high selectivity and efficient degradation of CDK2 (Wang et al., 2021). **CPS2** had good selectivity and wide application range, and we tried to test more than 10 cell lines and found it can effectively induce the degradation of CDK2 at nanomolar concentrations. We also used kinomics, kinase activity test, proteomics, and western blot experiments to have proved that the degradation of CDK2 was the most significant when **CPS2** was used to treat cells, while it had no significant effect on other proteins.

At the same time, we also found that under the treatment of **CPS2**, the differentiation index of AML cells was significantly increased, and the cell morphology was more mature. We conducted multiple sets of rescue experiments to have proved that **CPS2** performed differentiation-inducing function by the degradation of CDK2 in cells. In order to fully demonstrate the importance of **CPS2** as a selective degrader of CDK2 in the clinical treatment of AML, we collected multiple primary cells from AML patients and added **CPS2** for treatment. The results showed that the primary cells can also be significantly differentiated under the treatment of drugs, which fully proved the significance of **CPS2** potential in clinical application.

## Protac Provides a New Type of Rapid and Reversible Chemical Knockout Method

Using PROTAC to establish a protein knockdown animal model can be used as a powerful supplementary method for studying the loss of target gene functional sequence. The traditional genetic method is to establish animal models through genetic modification, such as TALEN or CRISPR-Cas 9. However, these technologies are often difficult to achieve rapid, efficient, and reversible protein degradation, and the long cycle and high cost bring more challenges to research, especially in non-human primates. At the same time, gene knockout models may exist potential gene compensation or gene mutations that led to phenotypic misunderstandings. In addition, the increased possibility of animals activating compensatory pathways may also obscure the phenotype. And right tools are still in urgent need for researches on those indispensable genes during embryonic development. As a new, rapid and efficient method of producing protein knockdown models, PROTAC can be used as an effective supplement to existing genetic tools. The following mainly introduces some PROTAC tool molecules developed by our group.

### PROTAC Provides Tools to Rapid Knockout of the FKBP12

FKBP12 (FK506 binding protein 12) is a type of protein that specifically binds to macrolide immunosuppressants **FK506** and **rapamycin**, and is widely expressed in mammals. This protein interacts with calcium channel receptor (Nissin receptors) and keeps the calcium channel in a stable closed state. When FKBP12 dissociates from RyRs, RyRs opens and releases calcium ions, thereby realizing the regulation of important functions of the body through the calcium signaling pathway (Andrus and Schreiber, 1993; Holt et al., 1993; Teague and Stocks, 1993; Huse et al., 1999; Wiedeman et al., 1999). One of the important functions of FKBP12 is to participate in the development of the heart and to play an important regulatory role in the phenotypic differentiation of cardiomyocytes, the formation of heart structures and the initiation of heart beats. The absence of FKBP12 in the embryonic heart can cause severe developmental ventricular defects, leading to embryonic death. However, the functional study of whole body knockout FKBP12 in the heart of adult mice is not complete, and there is no research on knockout FKBP12 in large animals.

**FIGURE 11:**
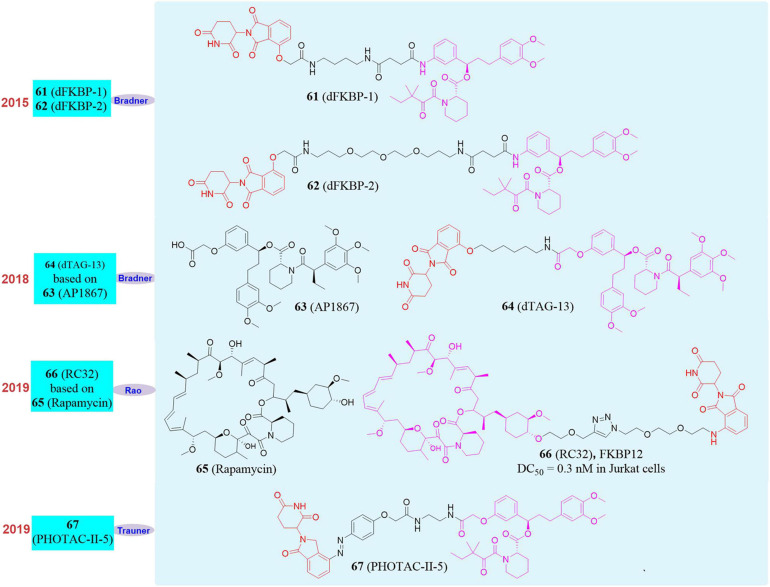
Structures of FKBP12 inhibitors and degraders.

In 2015, Bradner’s group reported the first PROTAC molecule targeting the degradation of FKBP12. They used CRBN ligand and FKBP12 wild-type inhibitor to design and synthesize two degraders, **dFKBP-1 (61**, Figure 11) and **dFKBP-2 (62**, Figure 11; Winter et al., 2015). These two molecules in human myeloid monocytic leukemia cell MV4-11 can well induce FKBP12 degradation at a concentration between 0.01 and 10 μM. However, they only verified that PROTAC can induce the degradation of FKBP12, but did not do further experiments to confirm the physiological function changes caused by FKBP12 degradation. Subsequently, in 2018, Bradner’s group reported a series of PROTAC molecules designed to target the degradation of FKBP12 based on the CRBN ligand thalidomide and FKBP12*^*F*36*V*^* selective inhibitor **AP1867** (Andrade et al., 2007; **63**, Figure 11). **dTAG-13** (Nabet et al., 2018; **64**, Figure 11) showed high selectivity and high efficiency to the degradation of FKBP12*^*F*36*V*^*. At the same time, they found that the constructed exogenous FKBP12*^*F*36*V*^* fusion protein FKBP12*^*F*36*V*^*-BRD4, FKBP12*^*F*36*V*^*-KRAS*^G12*V*^*, FKBP12*^*F*36*V*^*-EZH2, HDAC1-FKBP12*^*F*36*V*^*, MYC-FKBP12*^*F*36*V*^*, and PLK1-FKBP12*^*F*36*V*^* can also be efficiently degraded by **dTAG-13**. In addition, in a xenograft mouse model of MV4-11 cells stably expressing the luciferase-FKBP12 fusion protein, **dTAG-13** can also successfully induce the FKBP12 protein degradation in tumor cells. This dTAG technology not only reveals the physiological effects of BRD4 and KRAS*^G12*V*^* in detail, but also provides a new research strategy for the verification of targets in developing new drugs.

In 2018, our group successfully based on **Rapamycin** develop a small molecule **RC32** (Sun X. Y. et al., 2018, **66**, Figure 11; Saxton and Sabatini, 2017, **65**, Figure 11) that induces the degradation of FKBP12 protein. The whole body knockdown of the target protein in animals was established, and the whole body knockdown models of mice, rats, pigs and rhesus monkeys were quickly constructed. We also studied and verified the function of FKBP12 protein in mice and rhesus monkeys. Among them, FKBP12 protein in mice and rats, pigs, rhesus monkeys only take 1, 2, 3 days to knock down in the whole body with high efficiency. The conditional knockout of FKBP12 in the brain can be achieved when **RC-32** was administered to the cerebral ventricle. If the administration of **RC-32** was stopped, the protein in the animal body can gradually recover, which was conducive to the control of animal models and was more accurate for protein function research. Furthermore, this work verified for the first time that PROTACs can maintain high-efficiency protein degradation in the oral route of administration. We also applied this method to the systemic knockdown of other target proteins, such as BTK protein.

In 2020, Trauner’s group developed a series of light-controlled PROTAC for the first time, which was called PHOTOAC. They synthesized and screened the **PHOTOAC-II-5** (Reynders et al., 2020; **67**, Figure 11) by introducing the light control group azo in the linker of the PROTAC molecule, which showed effective degradation of FKBP12 between the concentration of 10 nM and 3 μM in the human acute lymphocytic leukemia cell line RS4;11 under the irradiation of light with a wavelength of 390 nm. The PHOTAC method precisely regulates the degradation of the target protein through optical control, thus providing a new strategy for photomedicine and photopharmacology. Therefore, the PROTAC is an effective supplement to the current gene knockout methods, and it is an extremely promising technology because of its rapid, efficient, reversible, and controllable realization of systemic protein knockdown *in vivo* and *in vitro*.

### PROTAC Provides Tools to Rapid Knockout of the HDAC6

HDAC6 (Histone deacetylase 6) is the most special histone deacetylase in the HDACs family. It is mainly located in the cytoplasm and the substrates include α-tubulin, HSP90, cortactin, etc. HDAC6 participates in the regulation of misfolded protein degradation, cell morphology and migration. More importantly, the abnormal regulation of HDAC6 is closely related to a variety of diseases, such as neurodegenerative diseases, cancer, and autoimmune diseases (Zhang et al., 2007; Simões-Pires et al., 2013; Krämer et al., 2014; Miyake et al., 2016). Therefore, directly regulating the protein level of HDAC6 is not only of great significance for disease treatment, but also has a profound impact on the biological process of its regulation.

**FIGURE 12:**
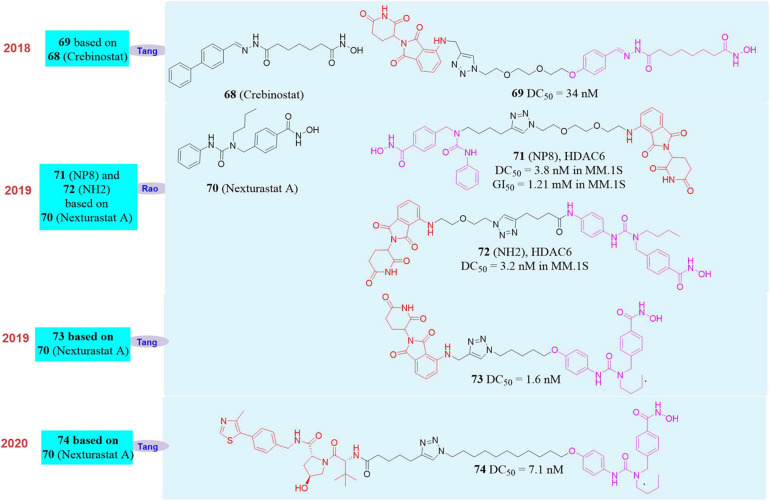
Structures of HDAC6 inhibitors and degraders.

In 2018, Tang’s group reported the first PROTAC molecule **68** (Yang et al., 2018; Figure 12) that induced HDAC6 degradation. This molecule was based on the broad-spectrum HDAC inhibitor **Crebinostat** (**69**, Figure 12) and pomalidomide as the E3 ligand. They found that it can selectively induce HDAC6 degradation with a DC_50_ of 34 nM, but had no effect on the other proteins in the HDACs family.

In 2019, our group used PROTAC to construct new HDAC6 protein degrader **NP8** (An et al., 2019; **71**, Figure 12) and **NH2** (Yang et al., 2019; **72**, Figure 12). Both **NP8** and **NH2** can be used in a variety of solid tumor cell lines such as human cervical cancer cells, human lung cancer cells, human glioma cells, human colon cancer cells, and hematoma cell lines (such as acute T cell leukemia cell lines, multiple myeloma cells). They can induce HDAC6 protein degradation in low-dose with high-efficiency, and reversible in those cells, among which the multiple myeloma cell line MM.1S was the most sensitive. In MM.1S cells, the DC_50_ of **NP8** to HDAC6 was only 3.8 nM, and the inhibitory activity of **NP8** and HDAC6 selective inhibitor **Nexturastat A** (Bergman et al., 2012; **70**, Figure 12) on MM.1S were equivalent. **NP8** was capable of inducing the degradation of the fusion protein of HDAC6 and EGFP, which proved that fluorescent methodology can be used to dynamically monitor the protein degradation process at the cell level, and provided a highly efficient and reversible tool for the research of HDAC6 protein. In the same year, based on **Nexturastat A** and pomalidomide as the ligands, Tang’s group also reported the HDAC6 protein degrader **73** (Wu et al., 2019; Figure 12), which can effectively induce HDAC6 protein degradation in a variety of tumor cell lines and the DC_50_ in the multiple myeloma cell line MM.1S was 1.7 nM. Compared with the inhibitor **Nexturastat A**, **73** had obvious anti-MM.1S cell proliferation function.

In 2020, Tang’s group reported another HDAC6 protein degrader **74** (Yang K. et al., 2020) based on VHL as E3 ligase and **Nexturastat A** as the protein binder (Figure 12). This molecule also can induce HDAC6 degradation effectively, and the DC_50_ in MM.1S cell is 7.1 nM. Based on the reports of Tang’s group and our group, it was found that PROTAC molecule based on broad-spectrum HDAC inhibitor such as **SAHA** can only selectively induce HDAC6 degradation, but did not have degradation activity to other members of HDAC family.

### PROTAC Provides Tools to Rapid Knockout of the HMGCR

**FIGURE 13:**
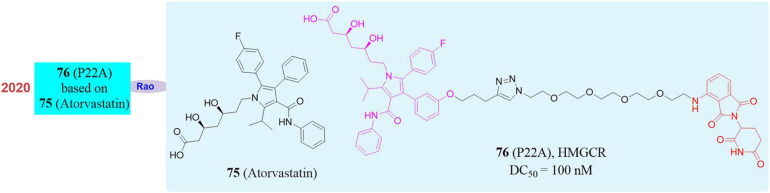
Structures of HMGCR inhibitor and degrader.

3-Hydroxy-3-Methylglutaryl Coenzyme A Reductase (HMGCR) is the rate-limiting enzyme in the cholesterol synthesis pathway. It consists of a transmembrane domain and a catalytic domain extending into the cytoplasm and locates in the endoplasmic reticulum (ER). Its main physiological role is to catalyze the conversion of HMG-CoA to mevalonate in the cholesterol biosynthesis pathway. Since the production of mevalonate is an irreversible process and inhibiting the activity of HMG-CoA reductase can hinder cholesterol synthesis, so it is one of the most important enzymes in the body’s cholesterol metabolism pathway and also a classic drug target for the treatment of dyslipidemia (Medina and Krauss, 2009; Clendening et al., 2010). In the market, HMGCR inhibitors are mainly statin type drugs. The mechanism is that statins have a HMG-like structure and will compete with the active site of enzymes to prevent the biosynthesis of mevalonate and downstream derivatives (including cholesterol) so as to achieve the effect of reducing plasma cholesterol levels, preventing atherosclerosis and treating cardiovascular diseases. Among all types of statins, **atorvastatin** (Cohen et al., 1999; Zeng et al., 2012; **75**, Figure 13) has shown excellent efficacy in reducing plasma low-density lipoprotein (LDL) cholesterol levels, and has achieved great success in clinical use to prevent and treat heart vascular disease. However, a considerable number of people are intolerant to statins, suffering from such serious side effects as skeletal muscle damage, which may be related to the increase in the compensatory expression of HMGCR in the body through negative feedback regulation after taking statins. Therefore, there is an urgent need for a strategy that can simultaneously eliminate HMGCR activity and abundance in clinical applications.

In 2020, our group successfully knocked down the HMGCR protein by using PROTAC. A series of small degraders were designed and synthesized based on atorvastatin as the target ligand and Pomalidomide and Lenalidomide as E3 ligands. Subsequently, *in vitro* degradation activity evaluation, it was found that the compound **P22A** (Li et al., 2020; **76**, Figure 13) induced HMGCR degradation in a dose-dependent manner, and reached up to 70% of the HMGCR protein degradation effect at 1 μM. When higher concentrations of **P22A** was used, no further degradation was detected. **P22A** had the best HMGCR degradation activity with a DC_50_ of 100 nM, which was significantly better than that of other compounds. In contrast, the inhibitor atorvastatin caused a significant upregulation of HMGCR. Besides, proteomics analysis found that the omics responses caused by the inhibitors **atorvastatin** and compound **P22A** were also very different. The inhibitors **atorvastatin** and compound **P22A** were comparable in inhibiting cholesterol synthesis and in the ability to up-regulate the expression level of LDLR induced by the SREBP pathway. Interestingly, HMGCR is an eight-pass transmembrane protein located on the endoplasmic reticulum. PROTAC had limited degradation ability of such proteins. This work proved the feasibility of using PROTAC to induce endoplasmic reticulum proteins degradation for the first time. In addition, the phenomenon of up-regulation of target proteins also appeared in many other inhibitors. This work showed that PROTAC had greater potential in application in this situation.

## Optical-Control Protacs Provide a New Type of Tool Enabling Reversible Activation and Deactivation of Protein Degradation

Although there are a large number of degraders and tool molecules that can quickly, reversibly and selectively down-regulate various proteins, scientists have little knowledge of the mechanism of degraders in biological systems and their deeper physiological effects. Therefore, the development of degraders with artificial controllability, high temporal and spatial selectivity, and simple control mechanisms is becoming more and more important. Once such degraders are available, scientists can not only learn more deeply in simple biological systems to understand the process and mechanism of protein degradation, it is also possible to explore the role of degraders in complex organisms by controlling the physiological activities. In order to achieve this, the method used must not cause interference to the biological system, and have the characteristics of high efficiency and simple operation (Brieke et al., 2012; Szymanski et al., 2013; Gautier et al., 2014). Therefore, light with high temporal and spatial resolution, which has been widely studied in neurobiology, chemical biology and disease treatment in recent years, appears in the vision of scientists (Madisen et al., 2012; Zhang et al., 2017; Tamura et al., 2019). Its extraordinary characteristics-high temporal and spatial accuracy, non-invasiveness, no interference to biological systems, and qualitative and quantitative control-coincide with the needs of scientists.

At present, two photo molecular biology methods with high temporal and spatial resolution have been widely used to study and control chemical and biological processes. The first method relies on the application of molecular photoelectric switches to perform isomerization between two or more states. This isomerization will lead to changes in molecular properties, which in some cases will be transformed into chemical or biological effects, and it is reversible. However, it is usually not enough to play a role in the major physiological activities of the research system. In this case, the second method, which is to use a photocleavage protecting group (PPG), is usually a better choice (Kessler et al., 2003; Stegmaier et al., 2008). This method can introduce the corresponding PPG into the structure of small molecules or proteins, and provide the corresponding molecule or protein activity through artificial light-induced cleavage of the protecting group (Hansen et al., 2015). This method has such advantages as good release performance and photo-cleavage, large amount of protective group and light source, non-toxicity, etc. However, since the cleavage of protective group is irreversible, this one is also an irreversible light-controlled biological method. On the basis of these studies, more and more scientists are focusing on the study of light-controlled small molecules to induce protein degradation.

**FIGURE 14:**
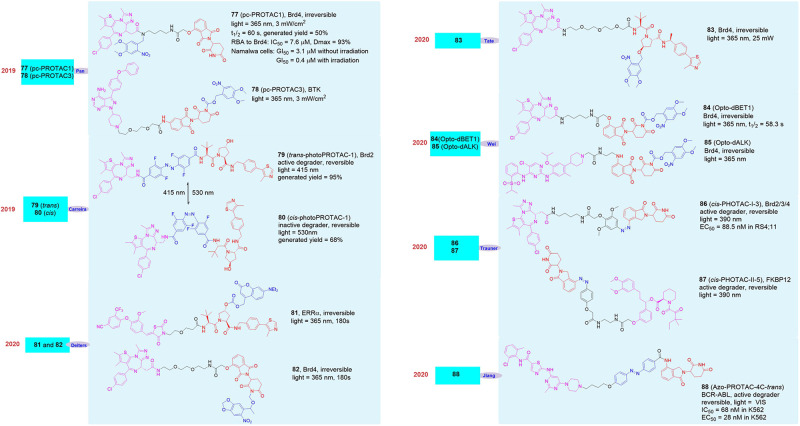
Structures of optical control degraders.

In 2019, Pan’s group reported the light-induced degrader **pc-PROTAC1** (**77**, Figure 14) designed based on the BRD4 degrader **dBET1** (Xue et al., 2019). They introduced 4,5-dimethoxy-2-nitrobenzyl (DMNB) groups in the linker of **dBET1**, which has made the newly synthesized degrader **pc-PROTAC1** have no physiological activity without light and has shown that it did not induce the degradation of BRD4 protein. However, when irradiated with a 365 nm light source, the degradation of BRD4 protein can be seen clearly when the drug concentration was 300 nM and its maximum degradation (D_max_) reached up to 93% at 1 μM. Although in the absence of light, its affinity for BRD4 protein was significantly weaker than **dBET1** (**pc-PROTAC1** was 7.6 μM vs. **dBET1** was 22 nM) and its inhibitory effect on cells was also significantly worse than **dBET1** (**pc-PROTAC1** GI_50_ = 3.1 μM vs. **dBET1** GI_50_ = 0.34 μM), its inhibitory effect on cells was equivalent to **dBET1** (**pc-PROTAC1** GI_50_ = 0.4 μM vs. **dBET1** GI_50_ = 0.34 μM) after light treatment. Later, they applied the molecule to zebrafish and found that **pc-PROTAC1** can produce the same physiological effects as **dBET1** did under light conditions. In order to further verify the rationality and universality of the strategy, they also designed and synthesized the light-controlled degrader **pc-PROTAC3** (**78**, Figure 14) for the BTK protein, and experimentally proved that the molecule can induce the degradation of the BTK protein under light conditions, which proved the practicability of the light-induced degrader release technology.

At the same time, Carreira’s group also reported a light-controlled PROTAC based on the BET protein degrader **ARV-771** (Pfaff et al., 2019). They analyzed the spatial configuration of **ARV-771** and introduced *ortho*-F_4_-azobenzene into the linker, which can transform into *cis* or *trans* under different light conditions. They synthesized the photo-control degrader **photoPROTAC-1** and it can effectively switch between the *cis* and *trans* configuration after 530 and 415 nm light irradiation. They found that under 415 nm light conditions **photoPROTAC-1** would transform into ***trans*-photoPROTAC-1** (**79**, Figure 14), in which form can induce BRD2 protein degradation. On the contrary, it will be converted to ***cis*-photoPROTAC-1** (80, Figure 14)under 530 nm light conditions, and this form will not induce BRD2 degradation.

In 2020, Deiters’s group also reported two cases of light-controlled degrader (Naro et al., 2020). The first was a VHL-based degrader **81** (Figure 14) targeting ERRα. They introduced diethylaminocoumarin (DEACM) through carbonate bonds in the degrader. The DEACM group can be activated under 405 nm light conditions, and then leave and peel off the degrader with biological activity, so as to realize the artificial controlled release of the degrader. The second example was the light-controlled degrader **82** (Figure 14) based on the BRD4 protein. They successfully introduced 6-nitropiperidinyloxymethyl (NPOM) into the degrader, of which the NPOM could generate photolysis reaction under 365 nm light condition. However, the activities of these two light-controlled degrader were obvious poor.

In addition to the above examples of light-induced degraders, other research groups have also reported similar molecules in 2020. As reported by Tate’s group, they introduced 4,5-dimethoxy-2-nitrobenzyl (DMNB) group into the VHL structure resulting in the light-controlled degrader **83** (Kounde et al., 2020; Figure 14), which achieved the successful release of the degrader under light conditions. Wei’s group reported that they also modified the CRBN structure with 4,5-dimethoxy-2-nitrobenzyl (DMNB) group, the BRD protein opto-PROTAC **opto-dBET1** (**84**, Figure 14) and the ALK protein opto-PROTAC **opto-dALK** (**85**, Figure 14) were successfully obtained, which achieved the successful degradation of Brd protein and ALK protein under light-controlled conditions (Liu et al., 2020). Trauner’s group introduced azo unit into degrader on the benzene ring in CRBN structure, the light-controlled reversible degrader ***cis*-PHOTAC-I-3** (**86**, Figure 14) and ***cis*-PHOTAC-II-5** (**87**, Figure 14) were obtained, which realized the light-controlled reversible degradation of BRD protein and FKBP12 protein (Reynders et al., 2020). Jiang’s group reported that the azo unit was introduced into the linker of the degrader, and the BCR-ABL light-controlled degrader **Azo-PROTAC-4C-trans** (**88**, Figure 14) was obtained, also it was proved that the light-induced degrader **Azo-PROTAC-4C-trans** had good biological activity in the cells (Jin et al., 2020).

**FIGURE 15:**
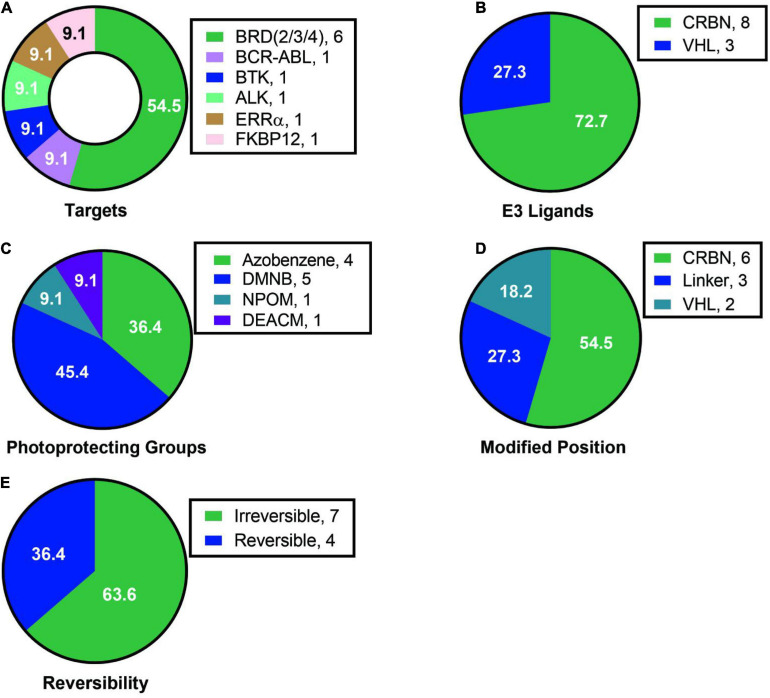
Summary of structure analysis of optical control degraders. **(A)** Targets used in loptical control degraders. **(B)** E3 ligands used in loptical control degraders. **(C)** Photoprotecting groups used in loptical control degraders. **(D)** Modified position in loptical control degraders. **(E)** The reversibility in loptical control degraders.

Analyzing the existing research on light-controlled degraders, we found the following characteristics: (1) Current researchers pay more attention to the mature targets for degraders, such as BRD protein degraders, which had 6 cases and more than half of them, and there are only a few cases for other protein targets (Figure 15A). (2) Among the light-controlled degraders, researchers prefers the structure containing CRBN (Figure 15B). The possible reason is that the synthesis of the degrader containing CRBN is simpler. However, whether the structure of CRBN and VHL in the degrader will affect the efficiency of light-controlled release and degradation activity is currently unknown, so further researches are needed to prove this. (3) The types of light-controlled groups currently are still in limited use (Figure 15C). Its use is mainly concentrated in the range of 365–450 nm, which makes this technology mainly be used in cells and cannot be used in live animals, thus limiting the promotion and application of this technology. Another important related issue that needs to be solved is whether we can find a suitable light control unit in the future to make it possible for fixed-point and timed release in living small animals. (4) The introduction of light-controlling groups is diverse, but in current studies researchers are more inclined to introduce the light control group into CRBN/VHL or linker, but not on the target protein ligand (Figure 15D). It is worth looking forward to whether there will be a breakthrough on this point in the future. (5) Both reversible degraders and irreversible degraders are available, and they have their own characteristics (Figure 15E). However, the currently available methods for reversible degraders are relatively simple, mainly based on azo groups, and there is also the problem of low conversion efficiency.

In short, PROTACs can not only be used as a potential candidate molecule for the treatment of diseases, but also as a tool molecule that can be precisely controlled on time and space scales. The above researches offer ideas for the development of PROTACs in the field of biology, further broaden the practical application of PROTACs, and provide tools for solving more unknown biological problems in the future.

## Conclusion

**FIGURE 16:**
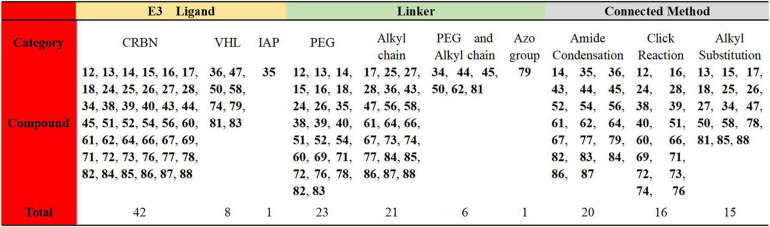
Summary and comparison of the 51 PROTACs mentioned in this review.

In this review, we have analyzed the related PROTACs that have been reported to illustrate their advantages from different perspectives. Due to the large number of targets and the complex structure of the PROTACs, 51 of them mentioned in this review have been summarized and analyzed. By comparing the types of E3 ligases, the linker types and connection methods, we have shown the results in Figure 16 below. It’s been found that the reported PROTACs were still diverse, but the E3 ligases used were mainly concentrated on CRBN and VHL. The linkers and connection methods were also mainly focused on PEG and alkyl. All of these gradually expose some challenges that currently exist in PROTACs.

The first challenge is about the rational design of PROTACs. For example, the human genome encodes more than 600 E3 ubiquitin ligases, but there are very few E3 ligases (VHL, CRBN, IAPs, and MDM2) currently used in PROTACs design. Therefore, how to expand the E3 ubiquitin ligase to be used in PROTACs is one of the challenges we are facing. In addition, how to expand the types of linkers and the connection methods are questions worth pondering.

The second challenge lies in the evaluation of the biological effects of PROTACs. Firstly, how to quickly and effectively screen the target protein ligand, evaluate the protein degradation activity and biological effects of PROTACs. Secondly, how to understand the relationship between degradation activity, selectivity and possible off-target effects (based on different targets, different cell lines, and different animal models). At last, PROTACs play a role in the catalytic cycle, and traditional methods cannot accurately assess the PK and PD properties of PROTACs; however, there is no mature PK and PD evaluation system. So, how to establish a scientific and credible PK/PD evaluation system is a question worth exploring.

The third challenge is mainly in the application of PROTACs. At present, there is only few cases of degrader reported for undruggable targets, and more cases are needed in the future to support the advantages of PROTACs in undruggable targets. It is reported that certain types of PROTACs can not only play a role through the mechanism of PROTAC, but also induce protein degradation through the mechanism of molecular glue. Therefore, how to distinguish the degradation mechanism (PROTAC or molecular glue or PROTAC and molecular glue) of proteins is also one of the challenges.

These are currently unclear problems that need to be resolved. What’s more, the above problems and challenges involve not only the technical application level, but also the basic fields, so it is necessary to strengthen communications from basic researches and application aspects. It is believed that through the joint efforts of colleagues in academia and industry, these questions can be answered satisfactorily in the near future.

## Author Contributions

YR designed the project, and reviewed and revised the manuscript. MH and WL summarized the literature, related structures and data. MH wrote the figures and manuscript. WL helped organize the manuscript. All authors contributed to the article and approved the submitted version.

## Conflict of Interest

The authors declare that the research was conducted in the absence of any commercial or financial relationships that could be construed as a potential conflict of interest.
